# Regulation of mammalian nucleotide metabolism and biosynthesis

**DOI:** 10.1093/nar/gkv047

**Published:** 2015-01-27

**Authors:** Andrew N Lane, Teresa W-M Fan

**Affiliations:** Graduate Center of Toxicology and Markey Cancer Center, University of Kentucky, Biopharm Complex, 789 S. Limestone St, Lexington, KY 40536, USA

## Abstract

Nucleotides are required for a wide variety of biological processes and are constantly synthesized *denovo* in all cells. When cells proliferate, increased nucleotide synthesis is necessary for DNA replication and for RNA production to support protein synthesis at different stages of the cell cycle, during which these events are regulated at multiple levels. Therefore the synthesis of the precursor nucleotides is also strongly regulated at multiple levels. Nucleotide synthesis is an energy intensive process that uses multiple metabolic pathways across different cell compartments and several sources of carbon and nitrogen. The processes are regulated at the transcription level by a set of master transcription factors but also at the enzyme level by allosteric regulation and feedback inhibition. Here we review the cellular demands of nucleotide biosynthesis, their metabolic pathways and mechanisms of regulation during the cell cycle. The use of stable isotope tracers for delineating the biosynthetic routes of the multiple intersecting pathways and how these are quantitatively controlled under different conditions is also highlighted. Moreover, the importance of nucleotide synthesis for cell viability is discussed and how this may lead to potential new approaches to drug development in diseases such as cancer.

## INTRODUCTION

A large fraction of the genome is now known to be transcribed into a wide range of RNAs whose functions are still being ascertained (1–4). Even in quiescent cells, there is considerable turnover of RNA involved in cell maintenance, repair and regulation. Proliferating cells must up-regulate RNA and DNA biosynthesis as an essential component of cell division, which can modulate, at least in part, the rate of the overall cell cycle (5,6). This requires increased expression of the genes associated with nucleotide synthesis in late G1 phase (5–15).

Nucleotide synthesis is regulated by several critical transcription factors (cf. Table 1), MYC and Rb/E2F in particular, which if mutated or overexpressed are associated with transformation and uncontrolled proliferation leading to cancer (16–23). MYC directly regulates the expression of genes that encode the enzymes in the nucleotide biosynthetic pathways and in the feeder pathways for the production of the precursors of all nucleotides (15,24–26), as well as coordinates RNA and protein biosynthesis (27,28). MYC also influences expression of specific microRNAs that regulate enzymes required for cell proliferation (22,29–31).

**Table 1.** Genes and enzymes involved in nucleotide biosynthesis

Gene name	Enzyme	Chromosome location	Reaction (Figure No.)	Predicted and known regulators
**1. Purines**				
*PRPS*	Phosphoribosyl Pyrophosphate Synthetase	Xq22.3	(Supplementary Figure S1)	GR Sox5 p53 FOXD1 Nkx2-5 FOXO3b POU2F1 POU2F1a FOXO4 GR-alpha
*PPAT*	phosphoribosyl pyrophosphate amidotransferase	4q12	1 (3)	Bach1, GATA-1, Bach2, C/EBPa, CHOP-10, Brachyury,Roaz, Arnt, USF-1; MYC
*GART*	*Trifunctional enzyme*. Phosphoribosylglycinamide Formyltransferase Phosphoribosylglycinamide Formyltransferase, Phosphoribosylglycinamide Synthetase; Phosphoribosylaminoimidazole Synthetase	21q22.11	2,3,5 (3)	SRY, HOXA9, HOXA9B, Meis-1, CUTL1, Stat5a, FOX O3/O3a/ O3b, MYC
*PFAS*	Formylglycinamide ribotide amidotransferase	17p13.1	4 (3)	AML1a,MAX,MAX1;MYC
*PAICS*	*Bifunctional* Phosphoribosyl aminoimidazole Carboxylase	4q12	6,7 (3)	AREB6, p53, CP2, STAT3, MyoD, MYC
*ADSL*	Adenylosuccinate Lyase	22q13.2	8 (3)	MEF-2,RORa2,
			12 (S2)	ARP-1,POU2F_1,1a,F2,F2B; MYC
*ATIC*	*Bifunctional* 5-Aminoimidazole-4-Carboxamide Ribonucleotide Formyltransferase/IMP Cyclohydrolase	2q35	9,10 (3)	FOXO1/a, C/EBPb,SREBP-1a,b,c, NFkB, AP-1, c-jun; MYC
*IMPDH1*	IMP dehydrogenase	7q31.3-q32	13 (3)	c-Fos USF1 AP-1 NRF-2 USF-1 c-Jun **MYC**
*GMPS*	GMP synthetase	3q25.31	13 (3)	E2F-4 E2F-3a E2F-5 E2F-1 E2F p53 E2F-2 C/EBPalpha
*ADSS*	Adenylosuccinate synthetase	1q44	11 (3)	USF1 Pax-5 NRSF form 1 USF2 CUTL1 NRSF form 2 Roaz FOXC1 STAT3 IRF-7A
*ADSL*	Adenylosuccinate Lyase	22q13.2	12 (S2)	
			8 (3)	
*AK1*	Adenylate kinase	9q34.1		c-Fos p53 AP-2alpha isoform 3 AP-1 AP-2alpha isoform 2 AP-2alpha isoform 4 c-Jun AP-2alpha AP-2alphaA
*NME*	Nucleoside Diphosphate Kinase	17q21.3		c-Fos AP-1 ATF-2 c-Jun

**2. Pyrimidines**				
*CAD, trifunctional*	Carbamoyl-Phosphate Synthetase 2	2p22-p21	1 (Figure 2)	PPAR-gamma1 AP-1 ATF-2 MyoD c-Jun PPAR-gamma2 CUTL1 ; **MYC, Hif1a, ER, SP-1**
	Aspartate Transcarbamylase		2 (Figure 2)	
	Dihydroorotase		3 (Figure 2)	
*DHODH*	Dihydroorotate Dehydrogenase	16q22	4 (Figure 2)	AhR AML1a p300 CUTL1 NF-kappaB POU3F2 Evi-1 Arnt GATA-2 NF-kappaB1 ; MYC
*UMPS*	*Bifunctional* Uridine Monophosphate Synthetase: orotate phosphoribosyltransferase	3q13	5 (Figure 2)	POU2F1a ER-alpha AML1a HTF AREB6 E2F E2F-1 POU2F1 POU2F1a ; MYC
*UMPS*	OMP decarboxylase	3q21.2	6 (Figure 2)	ER-alpha AML1a HTF AREB6 E2F E2F-1 POU2F1 POU2F1a
*NME*	Nucleoside Diphosphate Kinase	17q21.3		c-Fos AP-1 ATF-2 c-Jun ; MYC
*CTPS*	CTP Synthase 1,2	1p34.1		

**3. ribose/PPP**				
*G6PD*	Glucose-6-phosphate dehydrogenase	**Xq28**	(Figure 1)	TBP p53 ATF-2 c-Jun
*H6PD*	D-glucono-1,5-lactone lactone hydrolase (also G6PDH activity)	1p36	(Figure 1)	ER-alpha Spz1 NF-1 GCNF RORalpha2 Max GCNF-1 Ik-1 c-Myc
*PGD*	6-phosphogluconate dehydrogenase (deficiency not associated8 (3) with disease)	1p36.22	(Figure 1)	Bach1 Sox5 NF-1/L NF-1 HOXA5 NF-AT C/EBPalpha
*RPIA*	**ribose 5-phosphate isomerase A**	2p11.2	(Figure 1)	NMyc GR AML1a NCX/Ncx MyoD GR-alpha LCR-F1
*RPE*	**ribulose 5-phosphate epimerase**	2q32-q33.3	(Figure 1)	Sp1 AP-1 ATF-2
*TALDO1*	**Transaldolase 1**	11p15.5-p15.4	(Figure 1)	Pax-5 POU3F1 CUTL1 HNF-3beta YY1 AREB6 SRY FOXO4 FOXJ2
*TKT*	**transketolase**	3p14.3	(Figure 1)	NF-1 Sp1 p53 HFH-1 LUN-1 Egr-4 C/EBPalpha

**4. Feeder pathways**				
GOT1	Glutamic-Oxaloacetic Transaminase8 (3) (cytoplasmic)	10q24.1-q25.1		STAT1 STAT1beta Egr-4 STAT1alpha AREB6 Egr-2 PPAR-gamma1 FOXO1a PPAR-gamma2 FOXO1
GOT2	Glutamic-Oxaloacetic Transaminase (mitochondrial)	16q21		MIF-1 NF-kappaB GATA-2 AREB6 SRY POU2F1a NF-kappaB2 FOXJ2 (long isoform) FOXJ2 NF-kappaB1
GLUD1	Glutamate dehydrogenase 1	10q23.3		HOXA9B HOXA9 ER-alpha Elk-1 Pax-2 Pax-2a FOXJ2 (long isoform) ZIC2/Zic2 Meis-1a Meis-1
PHGDH	3-Phosphoglycerate Dehydrogenase	1p12	2 (Figure 4)	GR Max1 IRF-1 CUTL1 PPAR-alpha Max NRF-2 GR-alpha c-Myc
PSAT1	Phosphoserine Aminotransferase 1	9q21.2	3 (Figure 4)	POU2F2 (Oct-2.1) Oct-B1 oct-B3 oct-B2 POU2F2 POU2F2C POU2F1 POU2F1a c-Jun POU2F2B
PSPH	Phosphoserine Phosphatase	7p11.2	4 (Figure 4)	AREB6 GR CREB p53 deltaCREB SEF-1 (1) GR-alpha Nkx2-5
SHMT1	Serine Hydroxymethyltransferase 1 (cyto)	17p11.2	5 (Figure 4)	AhR CHOP-10 CBF-B CBF-A NF-YA c-Myb CP1A NF-Y CBF(2) C/EBPalpha
SHMT2	Serine Hydroxymethyltransferase 2 (mito)	12q12-q14	5 (Figure 4)	E2F-3a E2F-1 Sp1 E2F-2 GATA-1
TYMS	Thymidylate synthetase	18p11.32		AML1a p53 ATF-2 Egr-1 RREB-1 FAC1 POU2F1 POU2F1a ARP-1 MRF-2 MYC
DHFR	Dihydrofolate reductase	5q11.2-13.2		USF1 Sp1 p53 USF-1:USF-2 C/EBPalpha Pax-3 POU2F1 POU2F1a USF-1
MTHFD1	*Trifunctional*: Methylenetetrahydrofolate Dehydrogenase (NADP+ Dependent) 1, Methenyltetrahydrofolate Cyclohydrolase, Formyltetrahydrofolate Synthetase	14q24		RFX1 NF-YA NF-YC STAT5A CBF-C NF-YB HOXA5 CP1A CP1C NF-Y
RRM1	Ribonucleotide reductase subunit 1	11p15.5		E2F-4 E2F-3a E2F-5 E2F E2F-1 p53 HOXA5 Lmo2 E2F-2 ; MYC
RRM2	Ribonucleotide reductase subunit 2	2p25-p24		CREB AP-2alpha isoform 3 Sp1 AP-1 deltaCREB AP-2alpha isoform 4 AP-2alpha isoform 2 AP-2alpha AP-2alphaA

The gene nomenclature and regulation (including allosteric regulators) refer to mammalian systems. Predicted transcription factors are from http://www.genecards.org/, which recognize consensus binding sites in the promoters of given genes. Factors in b**old** represent those experimentally verified by at least one method. MYC binding to gene promoters is mainly from Liu (15); MYC regulation of TS, IMPDH2, PRPS2 is from (24) and E2F from (17). MYC regulation of nucleotide biosynthesis was confirmed by Kim *et al*. (28).

Despite its functional importance, nucleotide metabolism and cell cycle control have received much less attention than genomics and functional genomics, although there have been multiple metabolic targets derived from the relevant processes for human disease therapy, such as the antimetabolites MTX (32,33), gemcitabine (34), purine analogues (35), suicide inhibitors like 5-FU (36), a range of antiviral nucleotide analogues (37,38) and traditional RNA-seeking antibiotics (39).

Although numerous recent reviews have dealt with metabolic adaptations in proliferating cells (40–50), there has been little emphasis on nucleotide biosynthesis and its regulation. Here we review the regulation of energy and metabolic pathways needed for nucleotide biosynthesis in proliferating mammalian cells.

## CELLULAR CONTENT OF NUCLEOTIDES AND NUCLEIC ACIDS

### RNA and DNA content of mammalian cells

The DNA content of cells in an organism is fixed, and does not depend on the cell size. In contrast, other cellular components depend on cell volume, as the concentration is regulated. Thus, the protein concentration of mammalian cells is about 200 mg/ml (20% solution), which can occupy ∼16% of the cell volume, not counting the shells of ‘bound’ water in macromolecules. However, cell volumes vary widely - by more than an order of magnitude even for a given organism (see below). This means that the macromolecular content per cell with the exception of DNA varies over a factor of 10-fold or more from one cell type to another.

Quiescent mammalian cells in G0 or G1 are typically diploid, and contain the minimum amount of both DNA and RNA. In order to pass into S phase, the genes for DNA biosynthesis must first be upregulated. Furthermore, actively proliferating cells must double other macromolecular content as they enter M phase and divide into two daughter cells. As the major macromolecular component of cells is protein, protein biosynthesis must also be greatly upregulated during S phase. This will require an increase in the number of ribosomes and thus rRNA, as well as the concomitant energy production needed to meet the enhanced demand for the highly endergonic nucleotide biosynthesis (7,13,51). However, it has been argued that the utilization for macromolecule biosynthesis is a small fraction of the total cellular adenosine triphosphate (ATP) consumption (52,53). Nevertheless, the ATP concentrations (and probably the more relevant ATP/ADP.Pi ratio which is the thermodynamic potential (54,55)) cannot fall below critical levels to maintain cell viability. The ATP requirements show a hierarchy of proliferation > maintenance and repair > membrane potential (46,56–57), i.e. proliferation ceases first, while maintaining membrane potentials goes last. However in mouse Ehrlich Ascites cells, no such energy hierarchy was detected (58).

The content of mammalian cell biomass has been documented for different conditions (59,60). However, it is important to specify the cell type and conditions, whether it be cells in G0/G1 or proliferating cells, as well as the cell size. For example, all proliferating cells double their biomass as they complete a cell cycle. However, in the diploid state, the amount of DNA per cell is independent of the cell size, as it is fixed by the genome *C*-value (61). For human diploid cells, this is about 6.5 pg per cell based on 6 billion base pairs. However, cell volumes vary greatly, at least by an order of magnitude from small hematopoietic cells of 0.2–0.5 pl to large hepatocytes of volume >5 pl (60,62–63). As other macromolecules are maintained at about the same concentration per cell, the biomass content scales with cell size, as has been emphasized recently by Vaquez *etal*. (51).

Biomass accounts for around 30% of the cell volume (i.e. 70% water v/v). Given the relative abundances of the various macromolecules, their effective partial specific volumes and the density of mammalian cells, (64–66), (67) the biomass content of a cell is ca. 0.35–0.4 g/ml. In resting cells, biomass comprises ∼50–60% protein, 15% lipid, 5% carbohydrates, 5% small metabolites and ions, and the remainder the nucleic acids (i.e. ca. 15–20%). In a small cell, the DNA content may be up to 3% of the biomass, but in a large cell it is a much smaller fraction (cf. 0.2%). The remaining nucleic acid content is RNA, of which 85% is rRNA and <5% is mRNA. Mammalian cells have been said to have an RNA content of 3% of the biomass (68). However, this seems to be rather variable depending on cell type and size (58). Note that this is considerably less than the >20% estimated for *Escherichia coli* (52).

The RNA content of a cell does not necessarily represent the amount of RNA synthesis however, because the RNAs are synthesized as larger precursors that are then processed to the mature size, with recycling of the released mononucleotides. For example the ratio of intron to exon length in the human genome is about 28 (69) and the length of mature rRNAs are about 50% of the pre rRNA transcript (70). This requires at least three-fold more RNA synthesis than the amount of mature RNA present in the cell.

**Figure 1.**
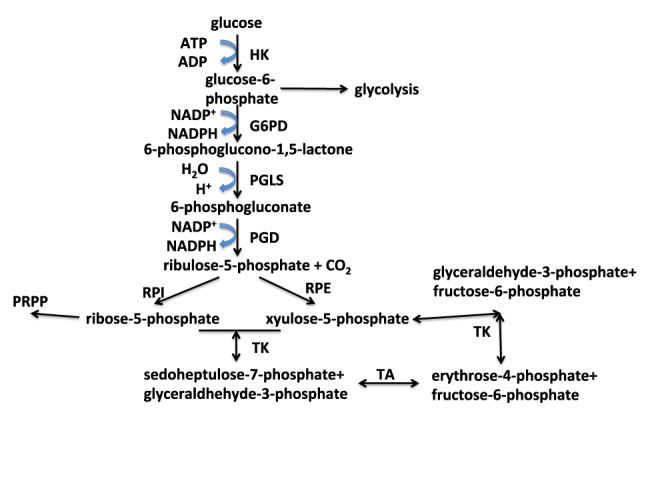
*De* *novo* nucleotide biosynthesis: generation of activated ribose. 5-phosphoribose-1-pyrophosphate (PRPP) is the activated form of ribose used for nucleotide biosynthesis and is derived from ribose-5-phosphate from the pentose phosphate pathway (PPP).Ribose-5-phosphate is produced via both oxidative and non-oxidative branches of the PPP. The oxidative branch also generates two NADPH. The oxidative branch comprises the reactions catalyzed by G6PD, PGLS and PGD. The non-oxidative branch interconverts five carbon sugars with four and six carbon sugars using the transaldolase (TA) and transketolase (TK) reactions. HK: hexokinase; G6PD: glucose-6-phosphate dehydrogenase; PGLS: 6-phosphogluconolactonase; PGD: 6-phosphogluconate dehydrogenase; RPI: ribulose-5-phosphate isomerase; RPE: PGLS 3-epimerase; TK: transketolase; TA: transaldolase.

Increased protein biosynthetic rate scales with cell size and progression into S-phase. To make protein faster, the cell must make more ribosomes, which requires more rRNA and therefore an increase in the rate of production of rNTPs, which suggests that RNA and protein biosynthesis are coordinately regulated. It has been shown that MYC and its downstream target translation initiation factor eIF4E achieve this via controlling the cis-regulatory element in the 5′ UTR of phosphoribosylpyrophosphate synthetase (PRPS2) (27), which catalyzes the first committed step in nucleotide biosynthesis (cf. Figures 1 and 3A).

### Cellular concentrations of rNTPs and ATP requirement for nucleic acid metabolism

To sustain energy and precursors for RNA biosynthesis, the ATP concentration in cells is generally maintained above 1 mM (71) with a high ATP/ADP.Pi ratio (72), so that there is sufficient free energy available to drive endergonic reactions. There are limits to the maintenance of these levels, below which different aspects of the cell functions cease, eventually leading to necrotic cell death (57,73–74). Some cells maintain very high concentrations of ATP (>5 mM) for specialized mechanical work, such as skeletal muscle (75) and cardiac myocytes (76). Similarly, other rNTPs are maintained at sufficient concentrations to activate and drive anabolic processes (see above) while supplying the materials for nucleic acid synthesis. Thus in cells, it is typically observed that the nucleotide concentrations are in the order ATP > UTP > GTP > CTP with the CTP level kept at ∼1 μmol/g (77,78). Nucleotide sugars are also needed to activate metabolites for various anabolic processes including UDP-glucose and other UDP-hexoses (for carbohydrates synthesis), CDP-choline (for lipid synthesis), GDP sugars (e.g. GDP mannose for glycosyltransferases), NAD(P)^+^,FAD/FMN (for mediating redox reactions) and ADP ribosylation for a wide range of regulatory functions (79,80). Collectively these compounds are typically present at >1 mM. For a cell of 1 pl volume, the amount of nucleotides is of the order 4–10 fmol. To maintain these pools at constant concentrations as a cell divides, these nucleotides must be synthesized.

For a cell to divide, the entire biomass including metabolites (which is cell size dependent) is doubled as it progresses from the beginning of G1 through the cell cycle and cytokinesis. As described above, a cell with a volume of 0.2–5 pl has a biomass content of 0.08–2 ng, comprising roughly 0.05–1.2 ng protein, 0.01–0.3 ng RNA, 6.5 pg DNA, 0.01–0.3 ng lipid and 0.004–0.1 ng each of carbohydrates and metabolites. The nucleic acids for cell doubling alone amount to 50–950 fmol nucleotides, which use eight ATP equivalents on average for *denovo* synthesis or 0.4–8 pmol ATP. However, as the RNAs are synthesized as larger precursor molecules and energy is required for recycling the released nucleoside monophosphate (NMPs), the actual ATP usage for RNA synthesis must be larger. For nucleic acid biosynthesis, which is energetically costly, the nucleotide synthesis consumes 0.5–9 pmol exogenous carbon, which is comparable in number to the ATP hydrolysis. Thus, to make nucleic acids for cell proliferation purposes, cells have to upregulate both energy metabolism and the nucleotide biosynthetic pathways. As expected nucleotide biosynthesis is greatly stimulated as cells enter rapid growth (7,11,16,81). Progression through the cell cycle is tightly regulated by numerous transcription factors, and is associated with changes in volume, energy and anabolic metabolism as cells progress through S-phase (15–16,49,51,62,81–87).

## NUCLEIC ACID SYNTHESIS: ENERGETICS AND NUTRIENT REQUIREMENTS

To maintain homeostasis, dividing cells need to replenish nucleotides at the same rate as cell division. Thus, the progression of the cell cycle must be tightly linked to the ability of the cell to acquire nutrients, generate metabolic energy and to drive anabolism, including nucleotide/nucleic acid biosynthesis. Although there are salvage pathways and cells can take up nucleotides (88–90), most proliferating cells synthesize nucleotides and nucleic acids *denovo*, mainly from glucose, glutamine and CO_2_. The metabolic demands of nucleic acid synthesis have been reviewed recently (47).

### Bioenergetics of nucleotide biosynthesis

**Figure 2.**
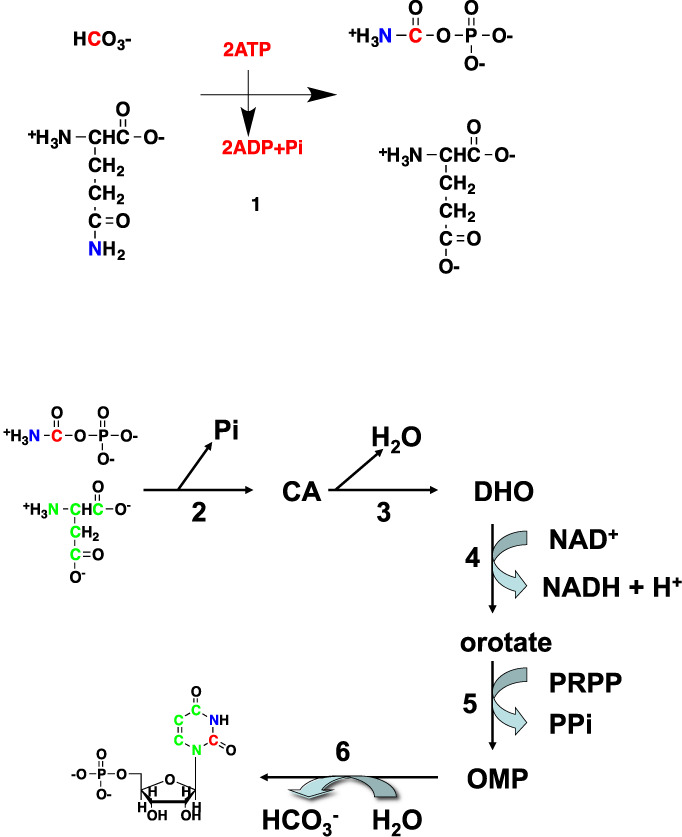
Pyrimidine biosynthesis. CA: carbamoyl aspartate; DHO: dihydroorotate; OMP: orotate monophosphate. Enzyme names: (**1**) carbamoyl phosphate synthase II (CPSII); (**2**) aspartate transcarbamoylase (ATCase); (**3**) carbamoyl aspartate dehydratase = dihydroorotase [*CAD* encodes enzymes **1** + **2** + **3**]; (**4**) dihydroorotate dehydrogenase; (**5**) orotate phosphoribosyltransferase; (**6**) orotidine-5-phosphate decarboxylase (OMP decarboxylase). The activities of **5** and **6** reside in a single bifunctional polypeptide encoded by the *UMPS* gene. Atom colors denotes origins: red from CO_2_, green from aspartate and ultimately glucose or Gln, blue from Gln.

**Figure 3.**
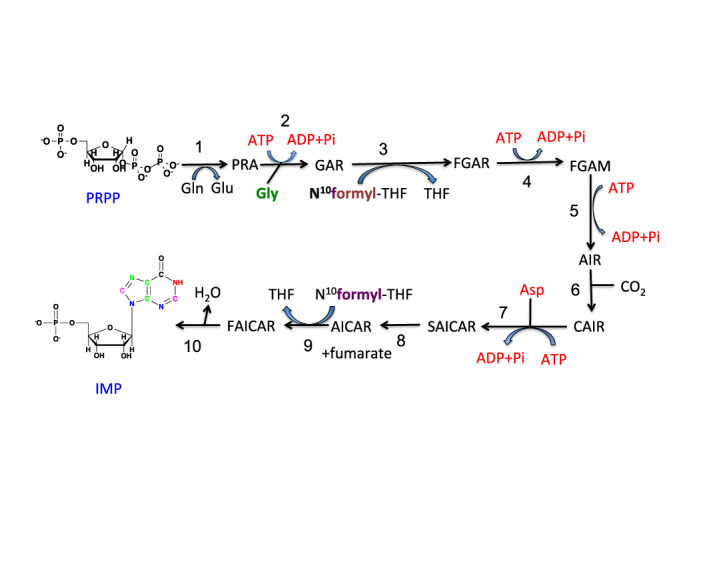
Purine biosynthesis: synthesis of IMP. Various atoms of the purine ring originate from different sources, i.e. N3, N9 derive from the amido group of Gln (blue), N7, C5, C4 derive from Gly (green), C6 from CO_2_ (black), N1 from the amino group of Asp (red) and C2, C8 from N^10^formyl-tetrahydrofolate. Enzyme names: (**1**) glutamine phosphoribosylpyrophosphate amidotransferase (PPAT); (**2**) glycinamide ribotide synthase (GART); (**3**) glycinamide ribotide transformylase (GART); (**4**) formylglycinamide synthase (PFAS); (**5**) aminoimidazole ribotide synthase (GART); (**6**) aminoimidazole ribotide carboxylase (PAICS); (**7**) succinylaminoimidazolecarboxamide ribotide synthase (PAICS); (**8**) adenylosuccinate lyase (ADSL); (**9**) aminoimidazole carboxamide ribotide transformylase (ATIC); (**10**) IMP cyclohydrolase (ATIC). IMP is the common precursor of AMP and GMP. The pathway from IMP to GMP and AMP are shown in Supplementary Figure S2.

The different parts of the nucleotides derive from various carbon and nitrogen sources in the cell (cf. Figures 1–3 and below), and the assembly of the mature rNTPs has a high metabolic demand. Supplementary Table S1 summarizes the numbers of nucleoside triphosphate (NTP) equivalents (number of phosphates released from ATP and GTP) needed to make one molecule each of the four rNTPs, according to Figures 1–3. Thus starting from glucose, three ATP equivalents are needed to make the activated ribose-5′-phosphoribosepyrophosphate (PRPP), which is produced by the reaction of 5′-phosphoribose with ATP, driven by the release of the good leaving group 5′-AMP (Supplementary Figure S1). The pyrimidine rings are synthesized first as uracil from aspartate, CO_2_ (or bicarbonate) and glutamine (Figure 2), which requires two ATP. One of the biosynthetic steps, i.e. orotate dehydrogenase reaction, occurs in the mitochondria (91), while the remainder reside in the cytoplasm. The amination of U to C consumes an additional ATP; *denovo* synthesis of 5′-UMP and 5′-CMP therefore require four and five molecules of ATP respectively. Aspartate provides three of the four carbon atoms of pyrimidines, which derive largely from glutamine and to a lesser extent from glucose (92) (and see below).

**Figure 4.**
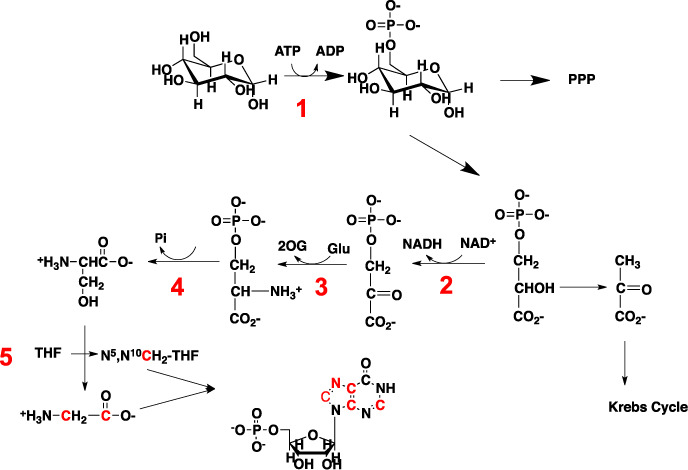
Glycine, serine and aspartate pathways. Synthesis of glycine and N^5^,N^10^-methylene tetrahydrofolate (N^5^,N^10^-CH_2_-THF) from glucose via the One-Carbon pathway. N^5^,N^10^-CH_2_-THF is further converted to N^10^-formyl-THF for incorporation into purine rings. *Enzymes*: 1: Hexokinase (HK); 2: 3-phosphoglycerate dehydrogenase (PHGDH); 3: phosphoserine aminotransferase (PSAT); 4: phosphoserine phosphatase (PSPH); 5: Serine Hydroxymethyltransferase (SHMT).

Unlike pyrimidines, the synthesis of purine nucleotides is entirely cytoplasmic, with the nucleobase being built directly on the activated PRPP (again, using three ATPs starting from glucose) (Figure 3A). The five carbon atoms of the purine ring derive from CO_2_, glycine and the one-carbon unit *N*^10^-formyl-TetraHydroFolate (THF), which is derived from the serine-glycine pathway via *N*^5^,*N*^10^-methylene-THF (Figure 4). The sources of serine and glycine may be exogenous, and/or via *denovo* synthesis from predominantly glucose (93–96). The production of the common intermediate in purine biosynthesis, Inosine Monophosphate (IMP), uses seven ATPs (Figure 3A; Supplementary Table S1). An additional GTP or ATP is used to convert IMP into AMP or GMP, respectively (Figure 3B). The NMPs are then converted to the triphosphate nucleotides by the action of nucleotide kinases, which use two additional ATP molecules for a total of 10. Hence for a genome of 50% GC content and 6 × 10^9^ nucleotides, DNA replication alone would consume around 6 × 10^10^ ATPs per cycle or close to 0.1 pmol ATP/cell and around 15 fmol glucose equivalents in terms of carbon. The energy demand for RNA synthesis may be 10-fold higher during a cell cycle, thus accounting for about 1 pmol ATP/cell.

The production of DNA further requires the reduction of rNDPs to dNDPs via ribonucletide reductase, which is NADPH-dependent (97), therefore requiring the supply of around 0.1 pmol NADPH/cell per division (and see below). The source of this NADPH is variable according to cell type (98), but the oxidative branch of the pentose phosphate pathway (PPP) is an efficient cytoplasmic source, which also links glucose metabolism to ribose production. As in general the DNA content of cells is lower than that of RNA, and compared with rNTPs, dNTPs have fewer functions other than nucleic acid synthesis, the concentrations of the dNTPs relatively low in cells, typically in the micromolar range in cells in G1, and rising ca. 5–10-fold in late G1 or during S phase (99,100).

**Figure 5.**
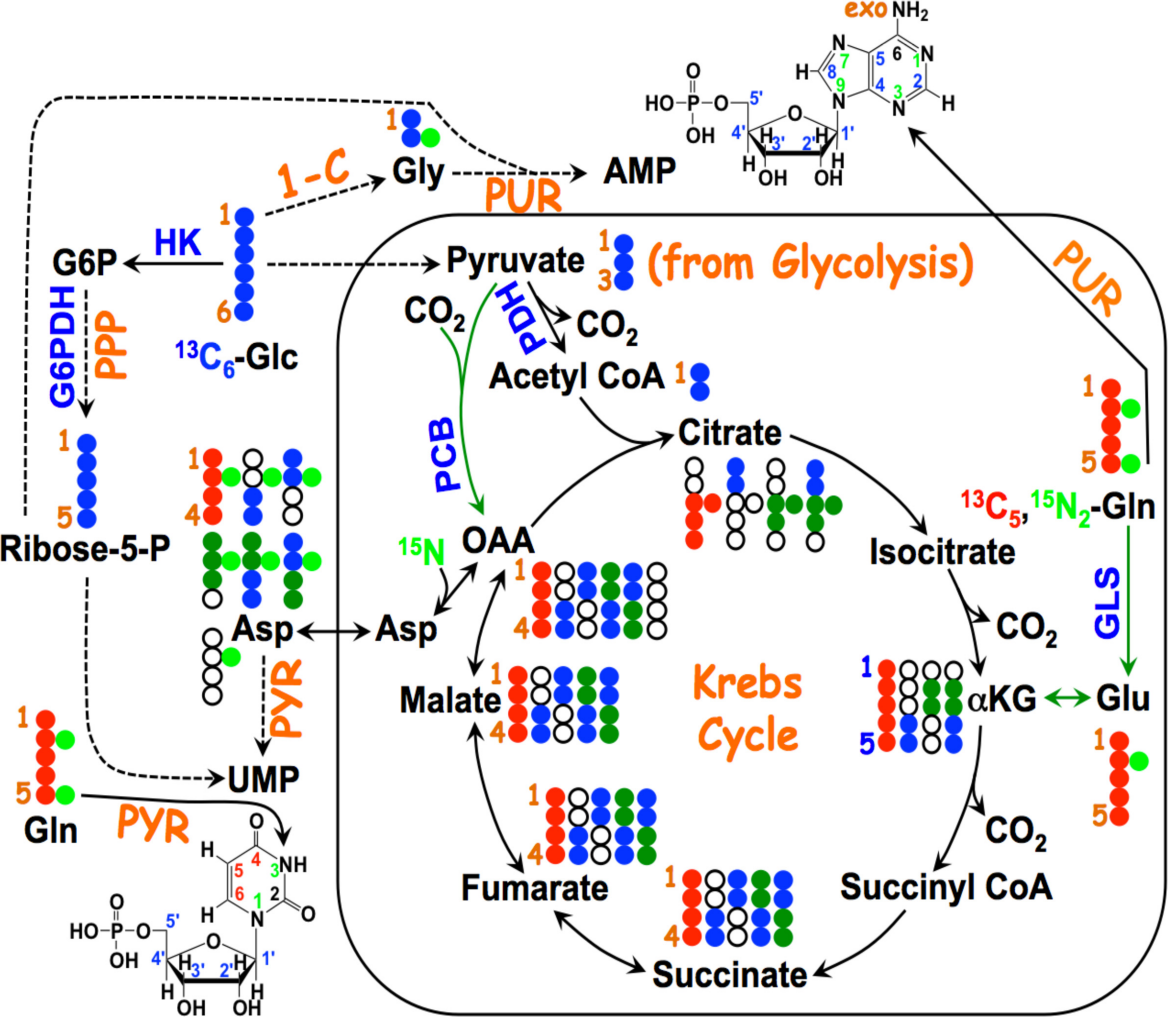
Atom resolved tracing from glucose and glutamine into ribonucleotides. The ^13^C labels from ^13^C_6_-Glc (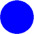) are incorporated into the ribose unit (via **PPP**), uracil ring (via the **Krebs cycle**–pyrimidine synthesis path or **PYR**) of UMP or adenine ring (via the one-carbon or **1-C** to purine synthesis path or **PUR**) of AMP (structures shown). The ^13^C (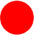) and ^15^N labels (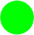) from ^13^C_5_,^15^N_2_-Gln are expected to go into the uracil ring (via the anaplerotic glutaminolysis or **GLS**-**Krebs cycle**-**PYR** path) of UMP and the adenine ring (via the **PUR** path) of AMP. The color of the label for atomic positions in the UMP and AMP structures is matched with that of ^13^C or ^15^N label derived from the glucose or glutamine tracer, except for C4-C6 of UMP where glucose or Gln-derived ^13^C is not delineated. Three examples of labeled uracil ring delineate contribution of ^13^C from ^13^C_6_-Glc or ^13^C_5_,^15^N_2_-Gln after one Krebs cycle turn without or with pyruvate carboxylation. The ^13^C labeling patterns of the Krebs cycle intermediates and Asp account for the ^13^C scrambling in succinate due to its symmetry and anaplerotic input (green arrows and 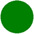) from pyruvate carboxylation into the Krebs cycle after the first turn. Open circles: ^12^C; HK: hexokinase; G6PDH: glucose-6-phosphate dehydrogenase; PDH: pyruvate dehydrogenase; GLS: glutaminase; PCB: pyruvate carboxylase; OAA: oxaloacetate; αKG: α-ketoglutarate; exo: exocyclic.

In addition to NADPH, as alluded to earlier, the nucleotide biosynthesis pathways have feeder pathways that provide for the carbon and nitrogen precursors, including the amino acids aspartate, glutamine, serine and glycine as well as CO_2_. These feeder pathways are glycolysis, the PPP (Figure 1), the serine-glycine pathways (Figure 4), the Krebs cycle without or with anaplerotic inputs (Figure 5) and glutamine amidotransferase reactions (Figures 2 and 3).

The metabolic energy needed to drive nucleotide biosynthesis is therefore substantial, and is expected to be derived from a combination of glycolysis and oxidative phosphorylation. The ATP yields for oxidation of different substrates assuming perfectly coupled mitochondria are given in Supplementary Table S2. Different cell types use different strategies that are also dependent on the tissue environment including the nutrient supply, which is a point of focus for understanding metabolic reprogramming in cancers (25,44,47,101–107). Although the oxygen concentration in tissues (e.g. solid tumors) can become quite low, reaching <1% or 10 μM (108,109), it is still sufficient to saturate cytochrome c oxidase, which has a low μM *K*_m_ for oxygen (110,111). However, other metabolic reprogramming occurs at oxygen levels lower than about 2–4%, due to the activation of HIF1α (112,113), which leads to accelerated glycolysis but inhibition of the Krebs cycle activity. HIF1α is constitutively degraded by the proteasome under normoxia (21% oxygen, ca. 210 μM in air-saturated water at 37°C) due to the regulation by HIF proline hydroxylase. However, when oxygen levels drop to below 2–4%, this enzyme activity is greatly attenuated (114), leading to HIF1α activation (112,115–116).

Moreover, the cellular respiration rate depends on cell type, the number of mitochondria present, the nutrient being oxidized (cf. Supplementary Table S2) and the overall metabolic demand. The oxygen consumption rate of some cancer cells has been measured as 4 fmol/min/cell under normoxia (21% oxygen), to 2 fmol/min/cell under hypoxia (1% O_2_) (117–120). Such cells can thus generate ca. 5 fmol ATP/min under 1% oxygen by oxidative phosphorylation. When compared with the nucleotide synthesis requirement during a cell cycle of ca. 1 pmol ATP, it would need about 3 h to produce this amount of ATP from oxidative phosphorylation to support nucleotide synthesis alone. However, hypoxic cells also ferment glucose to lactate, producing 2 ATP/mol glucose and in cancer cells glycolysis is typically accelerated many fold (42,121–122), which may produce ATP at a rate comparable to the more efficient mitochondrial oxidation of fuels including fatty acids (104,123), Gln (25,44–45,81,106,119,124–127) and ketone bodies (46) (Supplementary Table S2). It is notable that in the interstitial fluid of solid tumors, the glucose levels are very low with depletion of lipids compared with the blood supply in the tumor (128). This could reflect the high energy demand of tumor cells, which oxidize nutrients at a high rate.

### Enzymes and gene locations involved in nucleotide biosynthesis and ‘feeder’ pathways

Figures 1–4 and Table 1 lists the enzymes and genes directly involved in the *denovo* biosynthesis of nucleotides as well as the relevant ‘feeder pathways’ that supply the carbon, nitrogen and phosphorus, as well as metabolic energy. The genes are distributed over nine chromosomes for the purines and five for the pyrimidines, plus several others for the ‘feeder pathways’. The expression of the genes is thought to be controlled by several transcription factors, though relatively few have been directly demonstrated experimentally.

A notable feature of the enzymes involved in nucleobase biosynthesis is the multifunctional nature, including PPAT, GART and MTHFD1 for purines and carbamoyl phosphate synthase II (CPSII) aspartate transcarbamoylase (ATCase) dihydroorotase trifunctional enzyme (CAD) and UMPS for pyrimidines. These enzymes are thought to be rate limiting in nucleotide biosynthesis, though in practice this concept may not be helpful in understanding regulation (129–132). This is because a coordinate activation of multiple enzymes is necessary for enhancing flux through the pathway (131,133).

### Coordinate regulation of expression and flux control

The rate of cell growth is ultimately limited by the supply of materials and energy, and therefore by the flux through the relevant metabolic pathways. Nucleic acid biosynthesis, like any other complex intersecting metabolic networks requires coordination. The flux through even a simple linear pathway depends on the supply of the initial metabolite, the concentration and activities of all of the enzymes in the pathway, and the presence of any feedback or other regulatory controls. It is not well appreciated that the control of flux generally does not reside in any single enzyme or even the same enzymes under all conditions (129,131–134). Fundamentally, at steady state, the flux is the same at every point in the pathway. The concept of a rate-limiting enzyme is therefore at best conditional. In metabolic control analysis (MCA), the goal is to decipher how much control each enzyme in a pathway exerts on the net flux (which can be non-zero only where the system is not at equilibrium). A rate-limiting step in a multistep process is one that controls the net flux, such that changes in any of the other steps in the process have no influence on the rate. Glycolysis for example, is often considered to have three rate-limiting enzymes, HK, PFK-1 and PK. A single rate limiting enzyme is one that completely controls the pathways flux and its flux control coefficient (FCC) is unity. FCC is defined as ∂lnJ/∂lnv_i_ where J is the net flux and v_i_ is the enzymatic activity of the *i*th enzyme. If there are three equally rate limiting steps, then the flux control is shared equally among the three enzymes. As the sum of the FCC is unity, then each enzyme now has an FCC of one-third (131). In the context of MCA, this is equivalent to having the FCC of 0.33 for each of these enzymes, which is a form of distributed flux control. This means that changing the concentration of any one of these enzymes by 10-fold will change the flux by only a factor of 2, compared with a 10-fold change in flux if these enzymes were simultaneously changed 10-fold. In poorly vascularized tumors, however, the flux control resides mainly at the glucose uptake and HK steps (46,135).

Distributed control is a general feature of metabolic pathways. Even if there is one enzyme that is rate limiting, increasing its activity indefinitely will only transfer the rate limiting step to other enzymes in the pathway, thereby limiting the effect of up regulating a given enzyme activity (by expression or other enzyme-level activity control).

In pyrimidine biosynthesis, the rate limiting step has been described as CPSII (the first enzyme of CAD) (11) and dihydroorotate dehydrogenase (DHODH) (136), the fourth step in the pathway (Figure 2), again implying distributed flux control. This and the fact that several enzymes in both purine and pyrimidine biosynthesis have activities residing on a single polypeptide chain indicates a level of coordinate, stoichiometric expression and the possibility of channeling intermediates from one enzyme to another (see next section).

A means to coordinate expression of functionally linked enzymes that are coded for on different chromosomes is to make use of a common or a set of transcription factors. Although the promoters of the nucleotide genes have binding sites for a variety of transcription factors, there are many in common, one that stands out is MYC (along with related factors that interact with MYC (e.g. MAX and E2F), which have been independently verified by validation experiments such as MYC promoter occupancy assays (Table 1). The regulation of the expression of many genes by MYC is also linked to signaling pathways that respond to growth sensing cues, such as the EGFR/MAP kinase pathway (5,137–139), Hif1/2α (140–142) or estrogen receptor/Sp1 (137,143) for CAD regulation. Although not yet completely understood, cross-talk among these gene regulatory networks is likely to add additional complexities to homeostatic control.

In addition to transcriptional-level regulation, direct modulation of various enzyme activities of the pathways is important, as described below.

### Substrate level control

Several of the enzymes involved in nucleotide biosynthesis are regulated at the enzyme level by allosteric interactions, primarily by feedback inhibition. For example, the activities of the enzymes PPAT and CAD multifunctional enzymes are strongly inhibited by relevant pathway products (11,139). Purine biosynthesis is inhibited by AMP and GMP and Pi that act on PRPP synthetase (Supplementary Figure S1) (7) and by AXP and GXP (adenosine and guanosine mono, di or trihosphates) at two sites on the PRPP amidotransferase (Figure 3) (144–146). It is also evident that PRPP amidotransferase activity may be stimulated by the substrate PRPP (147). The branch point at IMP to AMP and GMP (Supplementary Figure S2) is further regulated by allosteric interactions, in which AMP and GMP inhibit the adenylosuccinate synthetase and IMP dehydrogenases, respectively (148–150). The enzyme activities therefore depend not only on the concentrations of the immediate substrates, but also on the end products. Substrate availability in eukaryotic cells can be an important means of controlling flux through pathways, either by physical compartmentation (important in pyrimidine biosynthesis) or by diffusion limits between different pools in large cells (151).

A potentially powerful means of overcome kinetic/diffusion limitations is to channel intermediates in multienzyme complexes (152,153). The observation that several of the enzymes in mammals are expressed as multifunctional enzymes (Figures 2 and 3, Table 1), and that these activities do not always catalyze sequential reactions has lead to the hypothesis that there might be a purinosome (91), in which intermediates are channeled rather than freely equilibrating with the bulk cytoplasm. Whereas the evidence for specific stoichiometric associations *invivo* is weak, kinetic experiments *invitro* are consistent with channeling, implying the possibility of weak and transient complex formation (154). Putative purinosome bodies have been detected *invivo* (91), but they may also be artifacts of the constructs used for visualization (155).

The pyrimidine synthesis pathway is controlled by feedback inhibition by UTP acting on the CPSII domain of the CAD trifunctional enzyme (Figure 2, Table 1) (5,11), which is also activated by PRPP, thereby integrating regulation of purine and pyrimidine biosynthesis (11). As for purine biosynthesis, mammalian pyrimidine biosynthesis is characterized by multifunctional enzymes (Table 1), in contrast to the homologues of *E*.*coli*, where CPS and ATCase are two non-associated polypeptides and are differentially regulated at the metabolite level (11). For efficient capturing of the CAD product dihydroorotate (DHO) by the mitochondrial DHODH, and the observation of the association of both CAD and mitochondria with the cytoskeleton, it has been suggested that CAD could be translocated via the cytoskeleton to the DHODH site (11). One complication is that up to 30% of the CAD may translocate to the nucleus during the S phase of the cell cycle, when demand for pyrimidine biosynthesis is maximal. This would compromise the DHODH efficiency and favor an alternative moonlighting function for nuclear CAD (11). However, DHO could still be readily diffuse from the nucleus into the mitochondria as the latter are in close proximity to the nuclear membrane (156).

### Synthesis of the deoxyNTPs

DNA synthesis requires a source of the four dNTPs, which derive from the ribonucleotides at the level of rNDPs by reduction at the 2′ position of the ribose subunit. The synthesis of dNTPs occurs cytoplasmically using the enzyme ribonucleotide reductase (RNR) (157), though the possibility of RNR activity inside mitochondria has been reported (158). This enzyme acts upon the rNDP and reduces the 2′-OH of ribose to the deoxy state, using NADPH as the electron source via thioredoxin and thioredoxin reductase. The resulting dNDPs are then converted to the dNTPs via dinucleotide kinase and ATP.

The DNA-unique nucleotide, dNTP is synthesized by methylation of dUMP by the action of thymidylate synthase using N^5^,N^10^-methylene tetrahydrofolate as the methyl carbon donor. dUMP is itself formed by a specific phosphatase activity on dUTP or by deamination of dCMP formed from hydrolysis of DNA (99). The dTMP is then converted to dTTP by the sequential action of two kinases.

The R1 subunit of RNR and TS are also regulated by MYC (Table 1). RNR expression is cell cycle dependent and as expected it rises during S phase (159,160). There is an alternative form of the R2 protein called p53R2 that is constitutively expressed at low levels but is upregulated in TP53(+) cells in the presence of DNA damage, and seems to be involved in DNA repair (161–163). The supply of dNTPs are moreover regulated at the substrate level by the supply of NADPH and allosteric interactions of RNR with ATP, dATP and the other dNTPs. RNR has two allosteric sites, both on the RRM encoded subunit. One binds either ATP (activating) or dATP (inhibitory). The ratio of the rATP to dATP therefore regulates net flux to dNDP according to demand—a high ratio of ATP/dATP favors a higher reductive flux and DNA synthesis, whereas a low ratio signals low demand for dNDP. The other allosteric site regulates substrate specificity (164). Thus binding of (d)ATP induces reduction of CDP or UDP, dGTP induces reduction of ADP and dTTP induces reduction of GDP. Together, these activities determine the supply of dNTPs during S phase (net DNA synthesis) and for repair synthesis.

## STABLE ISOTOPE TRACING OF NUCLEOTIDE SYNTHESIS

Although the metabolic pathways for nucleotide metabolism are well established, the detailed regulation at the enzyme (such as substrate channeling) and transcriptional levels under different conditions is less well understood. In order to define such regulation and the contribution of different nutrient sources for these complex intersecting pathways, it is necessary to measure the pathways in cells and tissues, using tracer technology such that individual atoms can be traced from the source to the product. In principle, radioisotopes can be used to trace precursors through metabolic pathways into final products (165,166). In practice stable (non-radioactive) isotopes offer more versatility and have the advantage of being fully compatible with live cells or tissues and making it possible to follow the fate of individual atoms from a precursor into detected products (167). The main analytical tools are mass spectrometry and nuclear magnetic resonance (NMR). Mass spectrometry distinguishes ^13^C or ^15^N from the more abundant isotopes ^12^C and ^14^N by virtue of the mass difference, which is nominally one mass unit. At sufficiently high resolution, such as FT-MS, differences in neutron mass can readily be detected. Thus the mass of a nucleotide containing one ^13^C atom versus one ^15^N atom differ by 0.00634 amu which is readily resolved at a resolution of 400,000 (168). This means that simultaneous dual labeling with both ^15^N and ^13^C can be used to determine the incorporation of the number and type of atoms (isotopologues) into nucleotides. NMR in contrast depends on the difference in magnetic properties of the nuclei of ^13^C versus ^12^C for example. ^12^C is magnetically silent, whereas ^13^C can either be directly detected or indirectly by its influence on the directly attached proton (92,169–170) which has the advantage of greatly enhanced sensitivity. NMR detects both the position (isotopomers) and the amount of incorporation of the labeled atoms. The analysis of the isotopomers and isotopologues of intermediates and products provides very detailed information about the biosynthetic routes to the products under study (25,167). Thus use of ^13^C and/or ^15^N labeled precursors coupled with labeled product analysis by MS and NMR offers the optimal approach for this purpose, as these techniques can directly detect the number and positions of ^13^C or ^15^N in the intermediates and products of nucleotide biosynthesis, thereby robustly defining the flow of atoms through the intersecting pathways. Other stable isotopes can also be used to measure nucleotide synthesis and incorporation into RNA and DNA. Hellerstein's group (14,171) has used both ^13^C glucose and ^2^H glucose with GC-MS detection of purine nucleotide released from DNA to follow DNA synthesis in cells and tissues. The overall turnover of nucleic acids in tissues can also be determined by measuring the incorporation of deuterons from D_2_O into DNA (172). Recently ^14^C-1 and ^14^C-6 glucose was used in comparison with [1–^2^H]-glucose or [3–^2^H]-glucose with LC MS to detect the transfer of a deuteron to NADP^+^ in the oxidative branch of the PPP (166).

Our group has developed the stable isotope resolved metabolomics or SIRM approach (173–175) for this purpose, which has been applied to mapping nucleotide biosynthesis in cultured cells or tissues *insitu* (19,92,101,168,173,176–177). Figure 5 tracks the expected incorporation of labeled atoms from ^13^C_6_-glucose (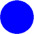,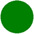 for ^13^C) and ^13^C_5_,^15^N_2_-glutamine (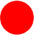 for ^13^C and 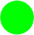 for ^15^N) into nucleotide products via purine (**PUR**) and pyrimidine (**PYR**) synthesis as well as feeder pathways including glycolysis, the Krebs cycle in the absence or presence of anaplerotic pyruvate carboxylation or glutaminolysis, PPP and one-carbon pathway. Based on SIRM profiling of the various labeled intermediates and products, these intersecting pathways for nucleotide biosynthesis can be rigorously reconstructed and quantified even in human subjects *insitu* (154). Examples of stable isotope tracer studies on nucleotide biosynthesis are described below.

### Tracking ribose synthesis via the pentose phosphate pathway

The ribose unit of the nucleotides derives from the PPP, and for most cells and tissues, this means that as few tissues are gluconeogenic the carbon originates from glucose either exogenous or via G-1P by phosphorolysis of glycogen. Two-dimensional (2D) NMR can be applied to crude cell or tissue extracts and discriminates the ribose subunit of purine and pyrimidine nucleotides as well as those of NAD^+^. The TOCSY experiment detects the scalar interactions between the ribose H1′–H2′–H3′–H4′ which appear as discrete cross peaks. When the carbon atom is ^12^C, the cross peaks are single, whereas if °C is present, it splits the attached proton resonance into two peaks separated by the one-bond coupling constant of 130–160 Hz. The patterns of thee so-called satellite peaks are characteristic according to which atoms are enriched in ^13^C, which gives rise to the isotopomer distribution that can be quantified (167,178). Complementary isotope edited experiments can be used to determine the complete isotopomer distribution in complex systems as needed (177,179). This is straightforward to demonstrate in the free nucleotide pool in cells by 2D ^1^H NMR analysis, as shown in Supplementary Figure S3. Here the ribose subunits of the free nucleotide pools are highly enriched with ^13^C when the cells are exposed to ^13^C glucose, but not at all when exposed to ^13^C Gln. The production of ^13^C_5_-ribose-containing nucleotides from ^13^C_6_-glucose, as determined by FT-ICR-MS analysis (**5**^13^C isotopologue in Supplementary Figure S4) is also consistent with the PPP activity The use of glucose as the primary carbon source for ribose biosynthesis applies to many different cell types (92,168,175,179–180).

However, it is difficult to delineate whether the ^13^C_5_-ribose labeling pattern results from oxidative, non-oxidative or both branches of the PPP. The oxidative branch (Figure 1) generates ribose-5-phosphate from glucose-6-phosphate (G6P) via decarboxylation of the C1 of G6P while producing two molecules of NADPH. This reaction is especially important in cells that have a high demand for NADPH, including erythrocytes (to remove H_2_O_2_ in a highly oxidizing environment (181)) and proliferating cells that are actively making fatty acids (166). The alternative non-oxidative branch reversibly converts ribose-5-phosphate and erythrose-4-phosphate into fructose-6-phosphate and glyceraldehyde-3-phosphate, and *viceversa* (182), which serves a dual purpose, namely to direct glucose carbon back into glycolysis and for nucleotide ribose biosynthesis. This branch does not generate NADPH. Boros *etal*. have established a simple GC-MS based stable isotope tracing method that can discriminate between these two branches of pathways, while estimating the net lactic fermentation flux (12,183–186). This method utilizes ^13^C_2_-1,2 glucose as tracer, which undergoes decarboxylation to become exclusively ^13^C-1-ribose via the oxidative branch of the PPP, whereas via extensive scrambling in the non-oxidative branch, ribose is labeled at the C1 and C2 positions. This scrambling arises from the reversibility of the reactions catalyzed by transaldolase and transketolase (cf. Figure 1A), which in fact may be exacerbated by the exchanges in the individual half reactions (187). Chemical exchange in reactions that are near equilibrium equilibrate label across the reaction, such that there can be label reaching a metabolite even where there is net flux in the opposite direction. This is a very general problem, that must be accounted for in any atom-resolved tracer experiments. The ratios of the different labeled ribose species provides an estimate of the relative flow through the oxidative and non-oxidative pathways subject to the caveats noted by Kleijn *et al*. (187). ^14^C-1,6 glucose has also been used to discriminate between the oxidative and non-oxidative branches, which relies on radiometric detection of CO_2_ released by the oxidation at C1′ (166). NMR analysis of free nucleotides can determine positional enrichment in the ribose subunits of intact nucleotides (92) without the need for fragmentation, which may provide a more direct discrimination of between the oxidative and non-oxidative branches and the relative flux through the two branches (188) (and A.N. Lane, unpublished).

It appears that both pathways may operate leading to net nucleotide ribose biosynthesis. Depending on the cell type, and possibly the growth conditions, the oxidative branch can account for very little to most of the flow of glucose into ribose (12,101,182–183,185–186,189–192). Such direct readout of the two pathway activities is required to determine the functional consequences of relevant gene or even enzyme expression in the two pathways (Table 1), as post-translational and substrate level regulations can further alter the metabolic outcome. Despite its complexity and importance to cell proliferation and maintenance, the flux through the PPP is only 1–7% of the net glucose flux to pyruvate (183,188,193–194).

### Tracking pyrimidine ring biosynthesis

The pyrimidine ring is synthesized independent of ribose-5-P, with all but one reaction occurring in the cytoplasm. The one exception is the DHODH reaction, which resides on the mitochondrial membrane and is intimately coupled to the electron transport chain (ETC). Mitochondria with a defective ETC cannot make UMP. As Figures 2 and 5 show, four of the ring atoms of uracil derived from aspartate, one from CO_2_ and the N3 from Gln via the cytoplasmic CPSII enzyme. Aspartate is a non-essential amino acid present in blood plasma at about 20 μM (195) and can be transported into the cell by Na^+^-dependent anionic transporters (196) or synthesized *denovo* by transamination of oxalacetate (OAA) in either the mitochondria or the cytoplasm. This reaction in fact is an important component of the malate/aspartate shuttle for transferring electrons from NADH in the cytoplasm into mitochondria. In addition to be the entry point of the Krebs cycle, OAA is the product of the anaplerotic reaction catalyzed by pyruvate carboxylase (PC), which is important in some cancers (179,197–198).

If aspartate and thus OAA is used for pyrimidine biosynthesis, anaplerosis is needed to replenish OAA to sustain the functioning of the Krebs cycle. In addition to PC, another common anaplerotic reaction in cancer cells and other proliferating cells (175,194) is glutaminolysis, in which glutamine is first hydrolyzed to glutamate and ammonia followed by either transamination with OAA to produce Asp + α-ketoglutarate (αKG) (199) or oxidative deamination via GDH to αKG + ammonium ions. αKG can also be converted to OAA by the normal functioning of the Krebs cycle (86,125–127,200–204) (Figure 5). The relative importance of these two anaplerotic reactions probably depends on cell type and growth conditions to maintain energy production, nitrogen balance and anabolic metabolism (175,179,194,198,205). The main difference between PC and glutaminolytic anaplerotic pathways is the production of ammonium ions by the latter, which in excess is toxic to cells and must be dealt with. Moreover, glutaminolysis competes with other Gln deamidation reactions as the direct nitrogen source for many anabolic processes including nucleotide synthesis (45,206–207). These considerations would probably in part govern the choice of anaplerotic pathways that proliferating cells adopt to meet their growth demand.

Again, tracer technologies can readily discriminate between glutamine and glucose as carbon sources and the different feeder pathways leading to uracil ring biosynthesis. [U-^13^C]-glucose enters the Krebs cycle via pyruvate either as ^13^C_2_-acetyl CoA (via PDH) or ^13^C_3_-OAA (via PC), which in turn gives rise to distinct labeling patterns in citrate, Asp and ultimately the uracil ring. For example, PC- (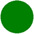) or PDH-derived ^13^C labeled Asp (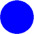) leads to the synthesis of ^13^C_3–_4,5,6-UMP or ^13^C_1–_6-UMP, respectively, after one Krebs cycle turn, as shown in Figure 5. Although [U-^13^C]-Gln also produces ^13^C_3–_4,5,6-UMP (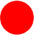) via the first Krebs cycle turn, the ^13^C labeling patterns of Glu, αKG and citrate derived from [U-^13^C]-Gln are distinct from those derived from [U-^13^C]-glucose (Figure 5) (25,81,92). Quantitative analysis of the isotope distribution in these intermediates and products provides a means of determining the relative contribution of Gln and glucose to uracil biosynthesis (92). It is generally found that in proliferating cells, whether transformed or primary cells that are stimulated to proliferate, Gln is the preferred carbon source for pyrimidine ring over glucose (92), but the precise balance is cell-dependent (Lane & Fan, unpublished data), and possibly also condition dependent.

Cells in culture synthesize a large fraction of the aspartate pool that is used for pyrimidine biosynthesis from glutamine and glucose (81,92,101,179,208), even in media such as RPMI that contain aspartate (0.15 mM) at concentrations much higher than that present in blood (ca. 20 μM). Also, in cells treated with ^13^C_5_,^15^N_2_-Gln, it is found by high resolution FT-ICR-MS that the aspartate pool is enriched mainly in the ^13^C_4_^15^N and ^13^C_4_^14^N isotopologues, despite the presence of high levels of exogenous aspartate (168). Both labeled Asp species are produced as a consequence of glutaminolysis and transamination. ^13^C_5_,^15^N_2_ glutamine produces ^13^C_5_,^15^N_1_ glutamate and then ^13^C_5_-αKG via glutaminase and aminotransferase activity. αKG enters into the Krebs cycle to produce ^13^C_4_-OAA, which is transaminated to form ^13^C_4_^15^N_1_-Asp or ^13^C_4_^14^N_1_-Asp in the mitochondria (Figure 5). The aspartate-malate shuttle then transfers the labeled Asp to the cytoplasm to supply both carbon (C4–C6) and nitrogen (N1) for pyrimidine biosynthesis. Thus, tracking the ^13^C and/or ^15^N fate from labeled precursors to the various intermediates and pyrimidine products enable a complete reconstruction of the feeder and pyrimidine synthesis pathways, including the delineation of different compartmental events.

However, there are few examples of this type of analysis in the literature, further studies are needed to determine whether other significant carbon sources are used by some cells (e.g. threonine in stem cells (209)) or under different growth conditions.

### Tracking purine ring biosynthesis

The *denovo* synthesis of the purine ring is considerably more complex than that of the pyrimidine ring, as the former is built up atom by atom on the phosphorylated ribose unit in the cytoplasm (Figure 3). The carbon sources are CO_2_, glycine and N^10^-formyltetrahydrofolate (Figure 4). Overall, glycine contributes up to four of the five carbon atoms in the purine ring, including indirectly via N^10^-formyl THF. Glycine is a non-essential amino acid that is present in human blood at ca. 0.25 mM (210) (HMDB: url-http://www.hmdb.ca/). It can also be synthesized from glucose via glycolytically produced 3-phosphoglycerate (3-PGA) (Figure 4), via the choline-betaine pathway (211) or from other sources such as threonine in some organisms, though not humans (212). It appears that the glycine precursor for purine biosynthesis is synthesized *denovo* from serine via glycolysis or mitochondrially via the glycine cleavage system (213) (Figure 4), and there may even be a net efflux of glycine in proliferating cells (214,215). In fact, exogenous serine, or endogenously synthesized serine, but not exogenous glycine is the major source of the one carbon units for purine biosynthesis (216). The nitrogen in purines derives from glycine (N7), glutamine (N3, N9) and Asp (N1) (Figure 3).

**Figure 6.**
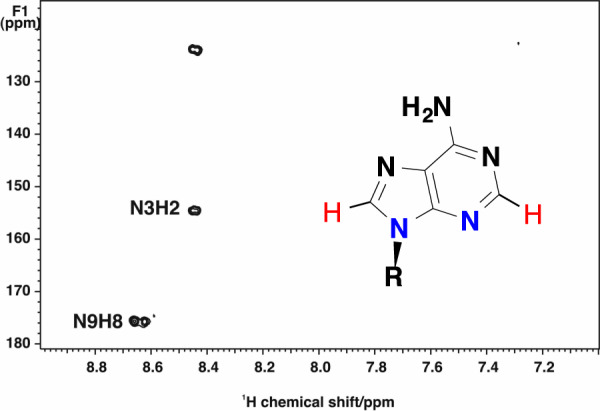
^15^N incorporation from [U-^13^C,^15^N]-glutamine into purines detected by HSQC. A549 cells were grown the presence of [U-^13^C,^15^N]-glutamine for 24 h. ^1^H{^15^N} HSQC NMR spectra were recorded at 18.8 T using an INEPT delay optimized for two bond couplings in aromatic systems. The two ring ^15^N atoms derived from the amido N of Gln (blue) are indirectly detected by their coupled protons (red) as cross-peaks of N3 to H2 and N9 to H8 in the adenine ring of AXP. Reproduced with kind permission from Springer Science+Business Media from J. Biomolec NMR (Springer) J. Biomol NMR. 2011 April; 49(3–4): 267–280. doi:10.1007/s10858-011-9484-6 (figure 9).

Incorporation of ring nitrogen atoms (N3 and N9) from the amido nitrogen of glutamine is readily observed in the free nucleotide pool using ^15^N-enriched Gln tracer coupled with 2D ^1^H(^15^N}-HSQC NMR analysis. The N3 and N9 of AXP and GXP can be detected in crude cell or tissue extracts using the 2-bond scalar coupling of N9 to C8H or N3 to C2H (cf. AXP data in Figure 6). This ^15^N incorporation pattern is evident in different cell types (19,92,177). In addition, using high resolution FT-ICR-MS, the incorporation of ^13^C and ^15^N from [U-^13^C,^15^N]-Gln into nucleotide can be simultaneously tracked by utilizing the difference in the effective neutron mass in ^15^N versus ^13^C. This capability makes it practical to reconstruct both carbon and nitrogen pathways without the need for separate experiments (168). Supplementary Figure S4 illustrates FT-ICR-MS analysis of the ^13^C labeling pattern of AMP derived from [U-^13^C]-glucose (168). Together with the NMR analysis for ^13^C labeled positions, we can ascertain that ^13^C_5_-AMP represents AMP with fully ^13^C labeled ribose subunit, while ^13^C_6-9_-AMP have fully ^13^C labeled ribose plus 1–4 ^13^C labels in the adenine ring (and see also (92,168,177). These data indicate that PPP, glycolysis, Ser-Gly-N^10^-formyl THF variably contribute to *denovo* synthesis of AMP. Thus, NMR and MS are synergistic in defining the source and detailed labeling patterns for nucleotides, from which the feeder and nucleotide synthesis pathways can be reconstructed and their modulation by transcription factors or environmental conditions can be elucidated.

Recently a tracer approach for following both NAD(P)H production from deuteriated substrates was used to determine the synthesis of glycine in cancer cells and it was shown that the serine/glycine pathway was a net producer of both mitochondrial glycine and NADPH (213,217).

### Salvage pathways

RNA in particular is constantly turned over in cells, both during the production of mature RNAs from longer precursors and to regulate the amounts of, e.g. mRNA. The breakdown of polymeric RNA and DNA results in release of NMPs, which can be recycled by the action of the nucleotide kinases. This process contributes to the nucleotide pools but cannot be used for net synthesis. Cells may also transport nucleobases from the external environment and add the appropriate sugar.

Addition of the nucleobase to ribose is achieved either via the PRPP step for purines, or catalyzed by specific pyrimidine phosphorylase to add the base to ribose-1-phosphate (218). Cytidine and deoxycytidine can be salvaged by the agency of cytidine deaminase, producing uridine or deoxy uridine, and then feed into the uracil pathway (219). This in essence randomizes the UTP/CTP pool and must be accounted for where isotope labeling experiments are performed. Cytidine can also be salvaged more directly via uridine-cytidine kinase producing (d)CMP (220). The relative importance of salvage versus *denovo* synthesis likely depend on the growth conditions and on the specific tissue. Recently it has ben argued that salvage pathways are very important in colorectal cancers for example, albeit without the assistance of isotope tracers (221).

### Tracking nucleotide incorporation into nucleic acids.

The free nucleotide pools in cells are straightforward to measure as they are relatively abundant, and are readily labeled using ^13^C glucose or ^13^C,^15^N glutamine (92,168,177,179). It is also practical to extract both DNA and RNA from ^13^C and/or ^15^N labeled cells and determine incorporation of exogenously supplied labeled tracers into nucleic acids (via free nucleotides) after hydrolysis to the NMPs (183,185). Although not all of the free nucleotide pool enters nucleic acids, it appears that there is a major common pool of labeled free nucleotides that is used for RNA synthesis (92).

### Tracking nucleotide synthesis *invivo*

The amount of *denovo* nucleotide biosynthesis correlates well with cell proliferation rates. On the other hand, tissues typically have a low mitotic index, even in tumors (which can be highly heterogeneous and may contain a rather low fraction of cancer cells) (222), so that the amount of *denovo* nucleotide biosynthesis is small. Nevertheless, using [U-^13^C]-glucose tracing in SCID mice, ^13^C incorporation into free nucleotide ribose was observed in some tissues (176), as well as in lung tumors resected from human subjects (175,223). The feeder pathways for nucleotide synthesis haves also been traced *invivo* in human glioblastomas using [U-^13^C]-glucose as tracer (194,198). ^13^C_2_-glycine was detected by ^13^C NMR in [U-^13^C]-glucose labeled glioblastoma tissues, demonstrating synthesis from glucose via the 3PGA-serine pathways (cf. Figure 4). This is despite the presence of 0.25 mM glycine in blood (210). In addition to the free nucleotide pool which is relatively well sampled by good extraction procedures (92,168,224), it is also possible to determine the enrichment in DNA and RNA (12,92,171–172,183,225). With modern instrumentation, the mass isotope ratios are generally good, at around 1% accuracy for medium resolution mass spectrometers, to <0.5% for high-resolution FT-MS instruments. Isotope accuracy in NMR is limited mainly by spectral quality rather than any instrumental issue, as the detectors are fundamentally linear over a dynamic range of ca. 2^20^. The major issues of accuracy and precision are related to experimental design and sample preparation. NMR methods do not require any fragmentation of the nucleotides, whereas GC-MS does. The experimental design must take into consideration multiple inputs to the nucleotide synthesis (see above), as well as turnover rates, which in tissues is complicated by the heterogeneity of cells, the efficiency of extraction and the generation of the nucleotides from the different kinds of polymeric material. Nevertheless, with careful consideration of the kinetics and of compartmentation, adequate models can be built that account for the observations without an excess of parameters, as shown for example for the synthesis of UDP-N-acetylglucosamine (173).

Thus, stable isotope tracers coupled with state-of-the-science NMR and MS analysis for labeling patterns of relevant metabolites provide an unprecedented opportunity for tracking nucleotide biosynthesis *invivo*, even in human subjects, under different tissue contexts and pathological conditions. These advances are expected to greatly accelerate our understanding in the regulation of human nucleotide metabolism, the perturbation of which is crucial to the pathogenesis of many human diseases, including cancer.

## CONCLUSIONS AND FUTURE DIRECTIONS

Nucleotides are used in a wide variety of metabolic function sin all cells, as coenzymes, for regulation, for activating substrates, for anabolic purposes as well as providing the subunits of the nucleic acids. Maintenance of nucleotide levels is therefore fundamental to cellular function. Proliferating cells, whether normal division during embryogenesis or in controlled proliferation in the hematopoietic system and stem cell compartments for example, as well as dysregulated cell division in cancer all need to maintain the supply of nucleotides. Unsurprisingly then the supply of nucleotides is strongly regulated and tied to the cell cycle. This is achieved both at the gene expression level by a variety of transcription factors as well as the substrates level regulation of the large number of enzymes needed to synthesize the nucleotides.

Although the basic nucleotide synthesis pathways are known and their energy demand can be estimated, the specific requirement for nutrient precursors/energy and the regulatory networks for modulating nucleotide biosynthesis in dividing cells remain unclear, particularly in terms of dependence on cell type and pathological conditions. This is in part due to the lack of powerful tools for elucidating the actual paths from nutrient precursors through various feeder pathways to *denovo* synthesized nucleotides. Furthermore, the amount of RNA synthesis greatly exceeds the amount of polymeric RNA present in a cell, owing to the extensive turnover. Characterization of gene and protein expression in these paths can provide useful clues, but such studies need to be validated by functional pathway analysis.

Stable isotope tracers in conjunction with state-of-the-science metabolomics methodologies is especially well suited for such functional studies not only in model cells and animals but also directly in human subjects. The relatively few stable isotope tracer-based studies reported have already uncovered important new insights into nucleotide biosynthesis, such as preference for endogenously synthesized precursors such as glycine and aspartate over those externally supplied, and how resources are reallocated according to the environmental conditions, especially in pathological conditions such as cancers (‘metabolic reprogramming’ (22,44)). Such information cannot be obtained without tracer methods. These considerations point to the need to be able to assess the nucleotide synthesis according to tissue pathology and nature, for which these new technologies can now be applied with some ease (176,194,197–198,226–227). It is notable that many drugs are targeted at nucleotide synthesis at the level of the availability of nucleotides or act as chain terminators (32–39,228–229).

In the future we expect further systems biochemical advances in systems biochemical approaches in which the detailed energy and anabolic pathways are integrated with the ‘feeder’ pathways and the specific gene expression networks that are linked to the cell cycle and thus nucleotide demand for DNA and RNA synthesis. As these are likely to be cell type and environment specific, the modem high throughput ‘omics approaches, such as SIRM described in this review are well suited to define the biochemical details of normal and pathological cell function, which directly informs the optimum modes for therapeutic intervention. As dysregulations of nucleotide metabolism are commonly involved in human disease pathogenesis, such understanding is expected to have important diagnostic and therapeutic benefits in relevant human diseases including cancer and diabetes.

## Supplementary Material

Supplementary Data
