# Supplementary material for: Regulation of mammalian nucleotide metabolism and biosynthesis
Source: Nucleic Acids Res. 2015 Jan 27;43(4):2466–85. doi: 10.1093/nar/gkv047 (PMC4344498; doi:10.1093/nar/gkv047)
Supplement: Supplementary Data [file gkv047_supplementary_data.zip › nar-02747-survey-d-2014-File004.pdf]

Table S1. ATP (GTP) utilization for de novo synthesis of rNTPs

Nucleotide	5'phosphoribosyl pyrophosphate from glucose	Nucleobase	rNMP	rNTP (total)
ATP	3	5	8	10
GTP	3	5	8	10
UTP	3	2	5	7
CTP	3	3	6	8

The numbers reflect ATP equivalents used to generate an rNTP de novo. The synthesis of AMP from UMP uses 1 GTP (cf Figure S2.)

Table S2. Maximum NTP yield from different substrates

Substrate	Process	Max ATP/mol substrate oxidized
Glucose	Fermentation	2
Glucose	Glycolysis	2
Glucose	Krebs Cycle oxidation of pyruvate/ox phos*	25
Glucose	Malate/Asp shuttle of NADH + ox phos*	5
Glutamine	Glutaminolysis → OAA: Krebs+ox phos	7.5
Glutamine	Glutaminolysis → mal → pyr: Krebs+ox phos [a]	17.5
Glutamine	Glutaminolysis → mal → lac: Krebs+ox phos [b]	5
Palmitate	β-oxidation+Krebs+ox phos	113
β-hydroxybutyrate	2AcCoA + Krebs+ox phos	21

*Oxidative phosphorylation, assuming P:O for NADH = 2.5 and 1.5 for FADH2 and 100% coupling

OAA: oxaloacetate; mal: malate; pyr: pyruvate; lac: lactate; AcCoA: acetyl CoA

A glutamine is oxidized via glutaminolysis through the Krebs cycle to malate (cf Fig. 5) and then oxidized to pyruvate by malic enzyme, and the pyruvate is then oxidized via the Krebs cycle.

B glutamine is oxidized via glutaminolysis through the Krebs cycle to malate (cf Fig. 5) and then converted to pyruvate by the malic enzyme; the pyruvate is then reduced to lactate via LDH

Figure S1: Synthesis of phosphoribosyl pyrophosphate (PRPP)

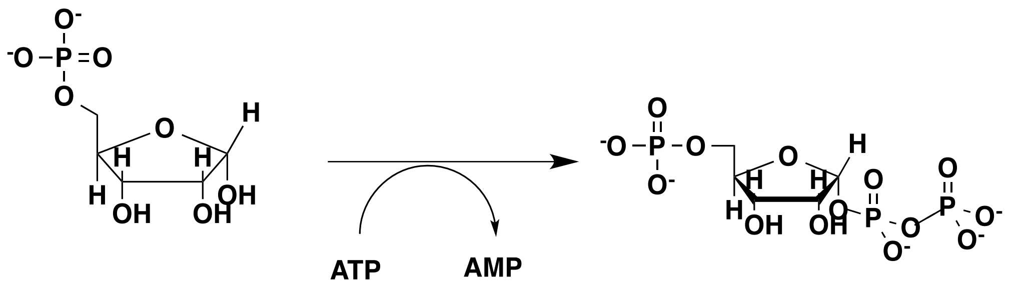

PRPP is generated from ribose-5-phosphate by the action of PRPP synthetase and requires energy in the form of ATP. Note that this reaction releases AMP, so two “high energy” phosphate equivalents are consumed per reaction.

Figure S2. Conversion of IMP (Figure 3) to AMP and GMP

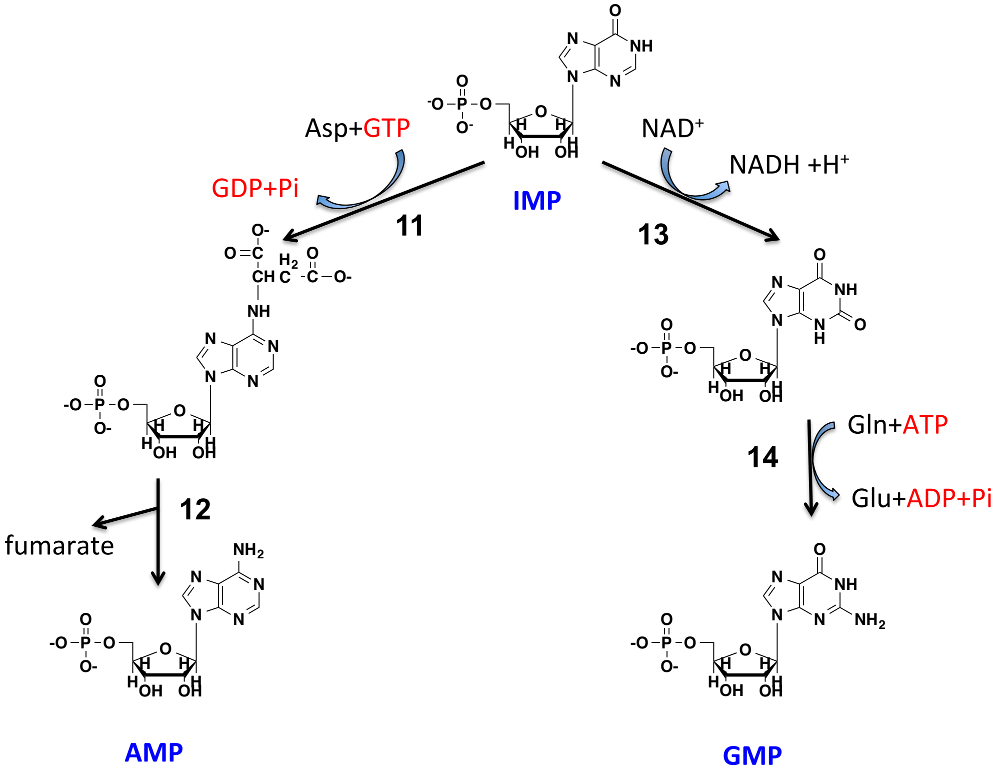

Enzyme names:

11. adenylosuccinate synthetase (ADSS)

12. adenylosuccinate lyase (ADSL)

13. IMP dehydrogenase (IMPDH)

14. GML synthetase (GMPS)

IMP is converted to AMP using the amino nitrogen of aspartate, and is driven by the hydrolysis of GTP. IMP is converted to GMP via an NAD-dependent oxidation reaction (13) and an ATP-dependent amination with nitrogen deriving from the amido group of glutamine (14)

Figure S3. 2D ^{1}H TOCSY analysis of a breast cancer cell extract shows incorporation of carbon from Glc and Gln into nucleotides.

A

B

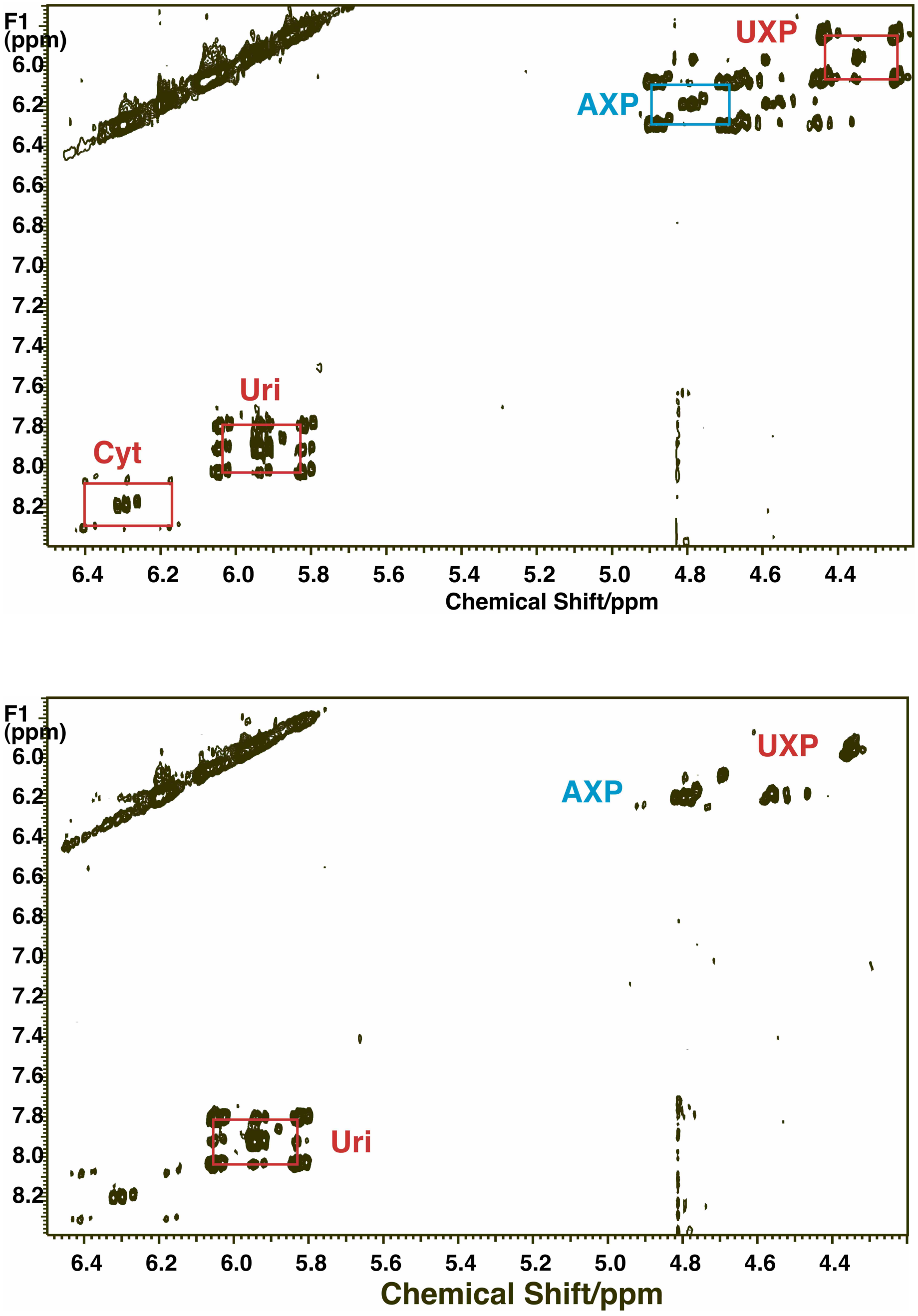

The estrogen-independent breast cancer cells MDA-MB-231 were grown in RPMI medium at 310 K in the presence of a 5% CO_2 atmosphere for 24 h in the presence of either 10 mM [U-^{13}C]-glucose, 2

mM ^{12}C Gln (A) or 10 mM ^{12}C Glc and 2 mM [U-^{13}C,^{15}N]-glutamine (B). After 24 h the cells were harvested and extracted with 10% trichloroacetic acid (TCA). The supernatant containing the polar metabolites was lyophilized and then redissolved in 100% D_2O containing DSS as a chemical shift reference and concentration standard (1). 2D ^1H TOCSY spectra were recorded at 14.1 T, 20 °C under standard conditions with a mixing time of 50 ms and a B_1 field strength of 8 kHz (2-5). The spectral region shown corresponds to the cross-peaks of H5, H6 resonances of the pyrimidine nucleotides (labeled Uri and Cyt) and those of the H1',H2' of the ribose subunits of the pyrimidine and purine nucleotides (labeled UXP and AXP). The boxes shown the ^{13}C satellites, whereas the central peak represent a proton attached to ^{12}C. Isotopomer analysis of the cross peaks and their identification was performed as described (6,7)

A. Using [U^{13}C]-glucose as tracer, ^{13}C enrichment is evident in both ribose and pyrimidine rings.

The labeling pattern of the uracil rings and ribose subunit differs for the two source molecules, reflecting the differential importance of glutamine and glucose input into aspartate via the Krebs cycle and into ribose via the pentose phosphate pathway (4).

B. Using [U^{13}C]-Gln as tracer, no ^{13}C is evident in the ribose rings, but strong ^{13}C enrichment is found in the pyrimidine rings.

Figure S4. **^{13}C incorporation from [U-^{13}C]-glucose into adenine nucleotides detected by FT-ICR-MS**

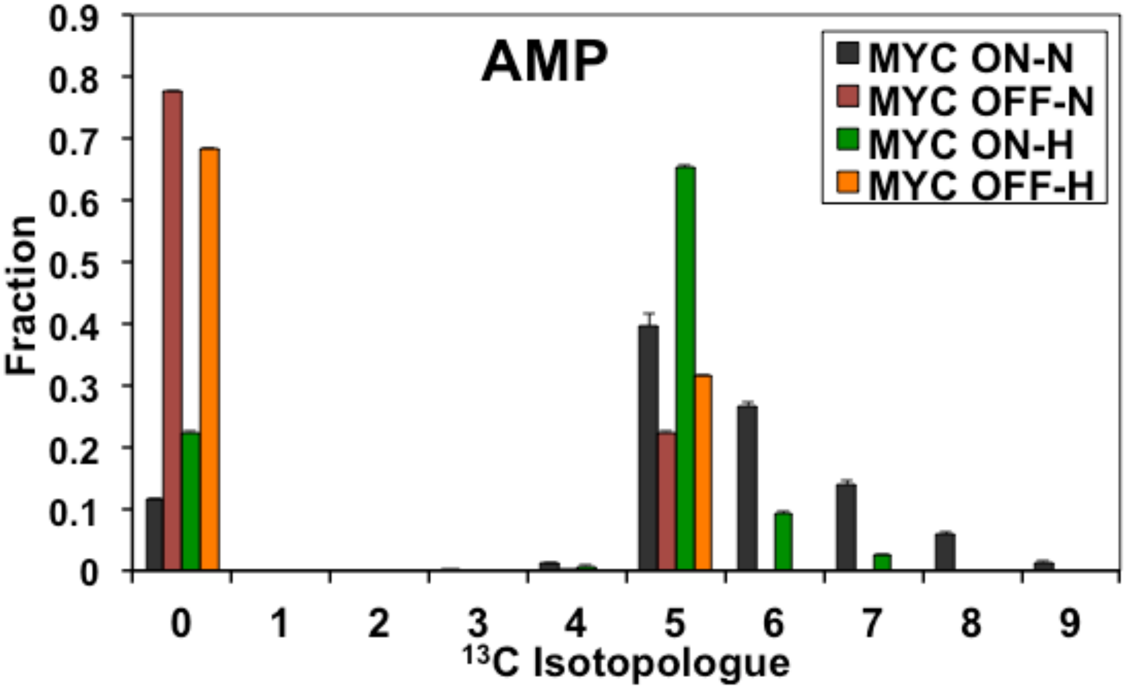

P493-B6 cells under a combination of basal MYC level (MYC OFF), MYC overexpression (MYC ON) normoxic (N) and hypoxic (H) conditions were grown in [U-^{13}C]-glucose for 24 h before extraction with acetonitrile:H_2O:chloroform (2:1.5:1, v/v), as described previously (5,8). The polar extracts were ion-paired with hexylamine before analysis by high resolution FT-ICR-MS (9). The fractional distribution of various ^{13}C isotopologues (**1** to **9**) of AMP were calculated after removing the natural abundance ^{13}C contribution (10,11) (12) and unpublished data from Fan, T. W-M, Le, A, Dang, C.V. and Lane, A.N.

Supplementary References

1. Fan, T.W.-M., Kucia M., Jankowski, K., Higashi, R.M., Ratacziak, M.Z., Ratacziak, J. and Lane, A.N. (2008) Proliferating Rhabdomyosarcoma cells shows an energy producing anabolic metabolic phenotype compared with Primary Myocytes. *Molecular Cancer*, **7**, 79.
2. Fan, T.W.-M. and Lane, A.N. (2011) NMR-based Stable Isotope Resolved Metabolomics in Systems Biochemistry. *J. Biomolec. NMR* **49** 267–280
3. Lane, A.N., Fan, T.W. and Higashi, R.M. (2008) Isotopomer-based metabolomic analysis by NMR and mass spectrometry. *Biophysical Tools for Biologists.* , **84**, 541-588.

4. Fan, T.W.-M., Tan, J.L., McKinney, M.M. and Lane, A.N. (2012) Stable Isotope Resolved Metabolomics Analysis of Ribonucleotide and RNA Metabolism in Human Lung Cancer Cells. *Metabolomics*, **8**, 517-527.
5. Fan, T.W.-M. and Lane, A.N. (2013) In Lutz, N., Sweedler, J. V. and Weevers, R. A. (eds.), *Methodologies for Metabolomics: Experimental Strategies and Techniques*. Cambridge University Press, Cambridge.
6. Lane, A.N. and Fan, T.W. (2007) Quantification and identification of isotopomer distributions of metabolites in crude cell extracts using 1H TOCSY. *Metabolomics*, **3**, 79-86.
7. Fan, T.W. and Lane, A.N. (2008) Structure-based profiling of Metabolites and Isotopomers by NMR. *Progress in NMR Spectroscopy*, **52**, 69-117
8. Fan, T.W. (2012) Considerations of Sample Preparation for Metabolomics Investigation. *Handbook of Metabolomics*, **17**.
9. Lorkiewicz, P.K., Higashi, R.M., Lane, A.N. and Fan, T.W.-M. (2012) High information throughput analysis of nucleotides and their isotopically enriched isotopologues by direct-infusion FTICR-MS. *Metabolomics*, **8**, 930-939
10. Moseley, H. (2010) Correcting for the effects of natural abundance in stable isotope resolved metabolomics experiments involving ultra-high resolution mass spectrometry. *BMC Bioinformatics*, **11**, 139.
11. Lane, A.N., Fan, T.W.-M., Xie, X., Moseley, H.N. and Higashi, R.M. (2009) Stable isotope analysis of lipid biosynthesis by high resolution mass spectrometry and NMR *Anal. Chim. Acta*, **651**, 201-208.
12. Fan, T.W.-M., Le, A., Stine, Z.E., Yang, Y., Zeller, K.I., Zhou, W., Ji, H., Higashi, R.M., Dang, C.V. and Lane, A.N. (2014) Antagonistic effects of MYC and hypoxia in channeling glucose and glutamine into de novo nucleotide biosynthesis. . *Cancer & Metabolism* **2** O10.
